# Molecular Biomarkers for Melanoma Screening, Diagnosis and Prognosis: Current State and Future Prospects

**DOI:** 10.3389/fmed.2021.642380

**Published:** 2021-04-16

**Authors:** Dekker C. Deacon, Eric A. Smith, Robert L. Judson-Torres

**Affiliations:** ^1^Department of Dermatology, University of Utah, Salt Lake City, UT, United States; ^2^Department of Pathology, University of Utah, Salt Lake City, UT, United States; ^3^Huntsman Cancer Institute, Salt Lake City, UT, United States

**Keywords:** melanoma, biomarkers, diagnostics, prognostics, likelihood ratio

## Abstract

Despite significant progress in the development of treatment options, melanoma remains a leading cause of death due to skin cancer. Advances in our understanding of the genetic, transcriptomic, and morphologic spectrum of benign and malignant melanocytic neoplasia have enabled the field to propose biomarkers with potential diagnostic, prognostic, and predictive value. While these proposed biomarkers have the potential to improve clinical decision making at multiple critical intervention points, most remain unvalidated. Clinical validation of even the most commonly assessed biomarkers will require substantial resources, including limited clinical specimens. It is therefore important to consider the properties that constitute a relevant and clinically-useful biomarker-based test prior to engaging in large validation studies. In this review article we adapt an established framework for determining minimally-useful biomarker test characteristics, and apply this framework to a discussion of currently used and proposed biomarkers designed to aid melanoma detection, staging, prognosis, and choice of treatment.

## Introduction

At a fundamental level, clinical tests are designed to aid clinicians in determining a best course of action when there is clinical uncertainty. These tests aim to improve certainty by ruling out a disease state, confirming a disease state, delineating prognosis, and/or identifying the most appropriate treatment regimen. Toward these goals, specific laboratory tests interrogate biomarkers: biological molecules that have clinically-relevant associations with a specific disease state. However, even a biomarker with an established disease state association may not be useful if it does not influence clinical decision-making. There are numerous factors that determine clinical utility: pre-test certainty of disease state, risks associated with making a clinical decision, cost, turn-around time, and the unique circumstances of each patient. Since biomarker development, from discovery to validation, requires substantial resources, investigators should carefully consider what characteristics are necessary for a useful clinical test at each stage of development.

With melanoma and other neoplasms, clinicians seek to balance the efficacy and potential side effects of an intervention with the estimated risk of disease progression. This risk has been traditionally inferred using prognostic tumor staging criteria, which takes into account tumor size, extent, nodal involvement, distant metastases, and histopathological grade and type. In more recent revisions of National Comprehensive Cancer Network (NCCN) and American Joint Committee on Cancer (AJCC) guidelines, select biomarkers have augmented traditional staging algorithms. The level of serum LDH is currently incorporated as a staging (prognostic) factor for malignant melanoma (1), and the mutational status of the *BRAF* proto-oncogene is predictive of therapeutic response (2–4). However, many critical intervention points in melanoma care remain fraught with uncertainty. Critical shortcomings in diagnosing, staging, and recurrence monitoring for melanoma could be alleviated with appropriate biomarker development. Toward this goal, many biomarker tests have been proposed, some of which are commercially available or are performed in academic referral centers. Presently, none of these tests have been compared to the current standard of care with large scale, randomized, prospective, multi-center, and independently validated studies with long-term clinical follow up.

For this literature review, we summarize the established theoretical framework for determining the minimal test characteristics required to potentially alter clinical decision making at different stages of melanoma care. We then apply this theory to evaluate currently utilized and proposed melanoma biomarkers. A recent review from the Melanoma Prevention Working Group critiqued the rigor in which commercially available prognostic tests have been validated and provided recommendations for care providers (5). Although we touch upon similar analyses here, our goal is to review a larger spectrum of biomarkers for melanoma care—preliminary to practiced—from the perspective of prioritizing further development. Given the substantial resources required for clinical test validation, we hope this discussion will aid the field in deciding which existing tests warrant further validation and which decision points are in most need of further biomarker discovery.

### The Case for Biomarkers in Melanoma Diagnosis and Treatment

Despite recent advances in treatment of advanced stage melanoma, particularly with the expansion of the use of immune checkpoint inhibitors (6), melanoma continues to confer significant morbidity and mortality. In 2015, there were 59,782 deaths attributed to melanoma worldwide (7). Nearly 1.6% of all cancer diagnoses are melanoma, and the disease accounts for 0.64% of cancer deaths (8). In the United States specifically, it is expected that there will be over 100,000 new diagnoses of melanoma in 2020 with almost 7,000 deaths attributed to melanoma (9). In Europe, while cost of treatment varies widely based on country and stage, melanoma treatment can range from several thousand to tens of thousands of Euros per patient on average, and these costs are expected to increase with wider adoption of immune checkpoint inhibitors and targeted kinase inhibitors (10).

Varied and unique uncertainties surround clinical decision-making during the detection, diagnosis, and treatment of melanocytic tumors. We have reviewed and analyzed four critical decision points during the identification and treatment of melanoma for which biomarkers that reduce uncertainty have been investigated. These decision points are: (i) deciding whether to biopsy a melanocytic neoplasm, (ii) confirming histopathologic diagnosis, (iii) stratifying risk for lymphatic spread with consideration for SLNB and, (iv) selecting systemic therapy. While this review focuses on cutaneous melanoma, the considerations discussed could be adapted to potential biomarkers for mucosal and uveal melanoma as well.

### Histopathology: the 14-Karat Gold Standard for Diagnosing Melanocytic Lesions

One poignant example of where biomarkers could dramatically enhance melanoma care is with the histopathologic diagnosis of melanoma. Histopathologic diagnosis is the gold standard for melanoma diagnosis, but, despite the advent of immunohistochemistry (IHC) and standardization of diagnostic criteria (11–13), it remains a subjective medical art constrained by significant intra- and interobserver variability. While nuances exist, melanocytic proliferations largely exist on a spectrum from histologically benign to malignant, as described in the MPATH-Dx Classification scheme (Table 1) (11). This classification scheme breaks melanocytic lesions into benign (class I), moderately dysplastic nevus and Spitz nevus (class II), severely dysplastic nevus, atypical Spitz nevi (class III), AJCC T1a and T1b invasive melanomas (class IV), and AJCC T1b-T4a invasive melanomas (class V). In the absence of metastasis identified during sentinel lymph node biopsy (SLNB) or further staging, these primary melanomas are considered Stage I-II using the AJCC 8th Edition Pathological Staging Criteria (14).

**Table 1:**
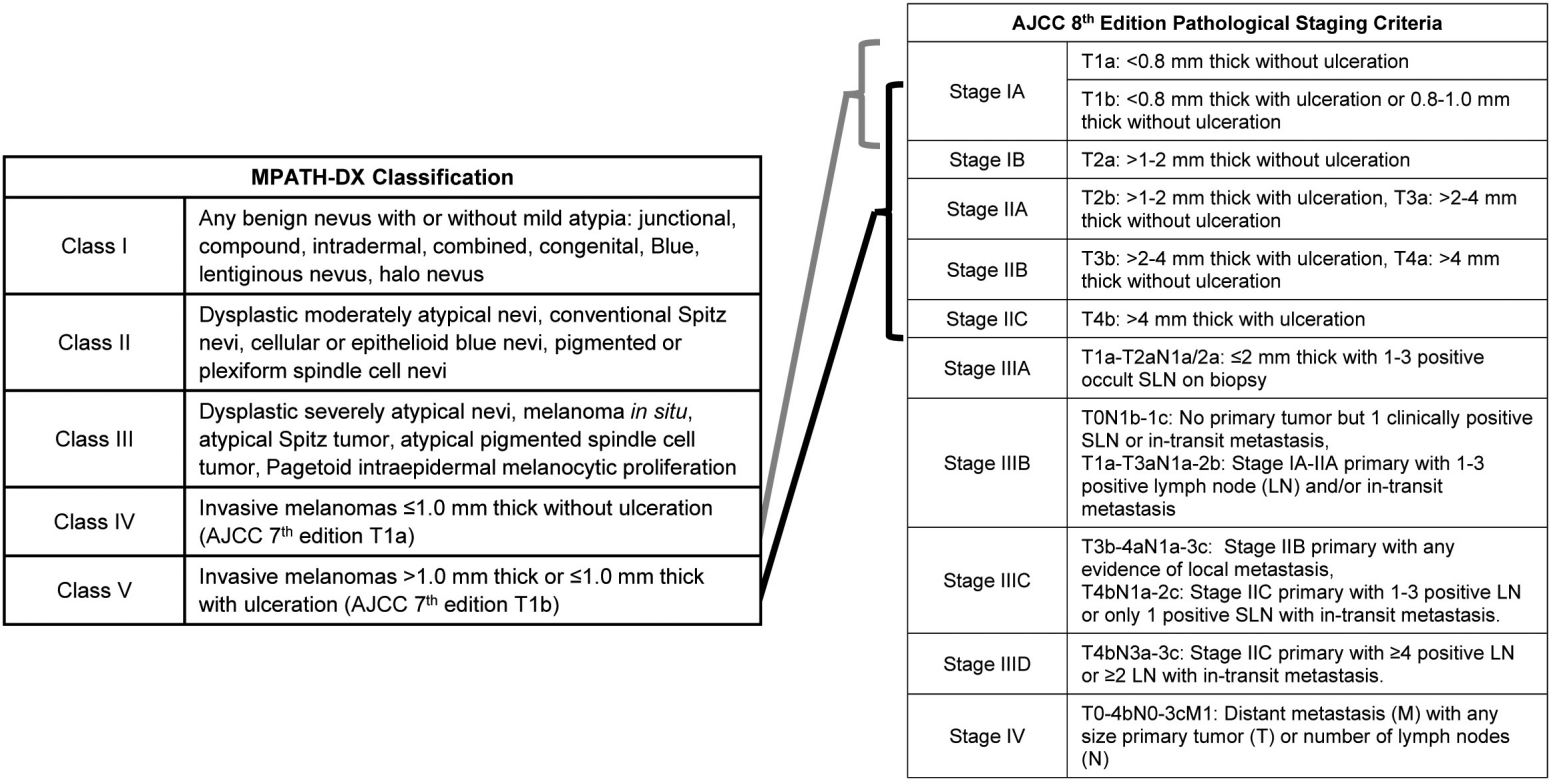
Abbreviated MPATH-DX classification and AJCC 8th edition staging criteria for cutaneous melanoma.

Although lesions at the extremes of the spectrum (class I and class V lesions) are most likely to be reproducibly diagnosed as such, there remains significant diagnostic discordance among dermatopathologists for each class. In one study evaluating 187 pathologists, MPATH-Dx class I and V have intraobserver concordance rates of 77 and 67%, respectively, and interobserver concordance rates of 71 and 55%, respectively. When compared to consensus diagnosis, the accuracies of individual pathologists were 92% for class I and 72% for class V (15). Separating class II, class III, and class IV is a greater diagnostic challenge. Class II have the poorest concordance (35% intraobserver, 25% interobserver) and consensus accuracy (25%), followed closely by classes III (60% intra- and 45% interobserver consensus with 40% accuracy) and IV (63% intra- and 46% interobserver consensus with 43% accuracy) (15). Similar results have been seen in smaller studies (16, 17). To summarize, these studies concluded that pathologists concurred with their own previous evaluations in only two-thirds to three-quarters of cases, and, for most stages, reached the same diagnosis as their colleagues less than half the time.

Given the poor concordance in distinguishing class II through IV melanocytic lesions, several reports have sought to identify barriers to reproducibility. Unsurprisingly, those pathologists who are trained in dermatopathology, have a significant caseload of melanocytic lesions, and have more than 5 years of post-training experience have better reproducibility and accuracy (18). Dermatopathology training reduced misclassification rates of class II lesions by 10% and class III-IV lesions by 20%. While second and third opinions from trained dermatopathologists can reduce the misclassification rate of a general pathologist, additional peer review has a smaller, but still significant, benefit when a dermatopathologist performs the initial read (19). If these findings reflect the pathology community, then three highly trained dermatopathologists reviewing the same class II, III, or IV case, would be expected to have a consensus accuracy of 35, 52, and 62%, respectively (19). These studies indicate that training and experience are not sufficient to reach high levels of accuracy for intermediate lesions. The most beneficial, albeit modest, improvement in concordance was obtained by updating from the AJCC 7th edition diagnostic criteria to the 8th edition (12), suggesting that further refinement of diagnostic criteria could yet improve accuracy. However, diagnostic threshold variability between observers and a tendency to over call diagnoses due to medicolegal concerns (diagnostic drift) are expected to limit the histopathological diagnosis of melanocytic lesions regardless of diagnostic criteria improvements (20–22).

### The Promise and Perils of Developing Biomarkers

The above observations highlight the need for objective biomarkers to improve diagnostic accuracy. Biomarkers that bring clarity to the MPATH-Dx classifications would improve patient care by more accurately matching intervention risk to lesion prognosis and save patients and medical systems from expense and potential adverse outcomes related to unnecessary procedures. However, the poor concordance and accuracy of histologic melanoma diagnosis highlight the substantial challenge in developing such biomarkers. The development and validation of clinically useful biomarkers require a gold standard, or a true state, for comparison. If only unambiguous cases are selected for study, then the biomarker, by definition, provides no clinical value to augment current standard of care. Alternatively, if the gold standards are sufficiently ambiguous (for example, a consensus accuracy of 35%), then a theoretically perfect biomarker is expected to fail validation. Thus, for prospective biomarkers to yield useful test characteristics and address a clinically useful decision-making process, careful selection of the gold standard is required. In addition to validated clinical utility, practical considerations such as cost, invasiveness, tissue requirement, and ease of performing the test may influence biomarker use by clinicians.

The resources required to validate a candidate biomarker are substantial in terms of cost, time, and, most critically, patient volunteers. For example, an international coalition of melanoma experts reported that a randomized clinical trial evaluating the added value of prognostic gene-expression based biomarkers would require cohorts of 1,000 to 9,000 patients, depending on the trial design and hypothesis being tested (5). Therefore, it is critical to carefully consider all of the potential pitfalls of biomarker discovery, development, and implementation prior to committing limited resources to validation of any specific test. Considerations include not only the performance of a proposed biomarker, but also the size and diversity of the cohorts from which it was discovered and validated, and its potential to integrate with current practice and positively influence clinical decision-making. Prior to development, investigators should consider what test characteristics are required for a test to be useful, so that the field can determine how best to distribute limited resources for biomarker discovery and validation. These characteristics are described next.

## Application of Fundamental Theory of Biomarker Utility To Current Melanoma Biomarkers

### Clinical Uncertainty in Melanoma Diagnosis and Treatment

**Figure 1:**
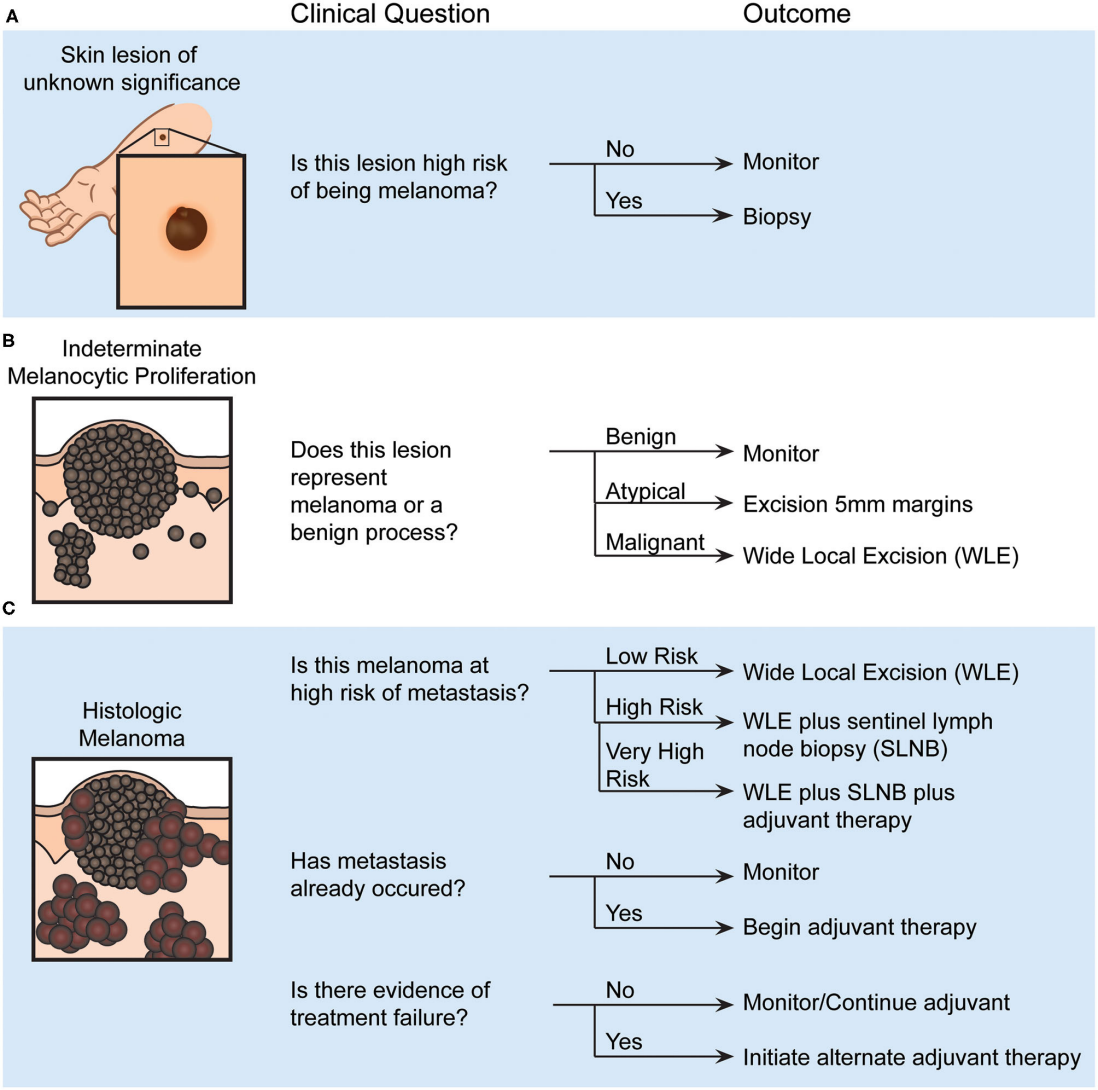
Examples of pigmented skin lesion clinical decision points and their outcomes, which could be aided by validated biomarkers. **(A)** Assessing a melanocytic lesion of unknown significance will result in a decision to either monitor or biopsy that lesion. **(B)** After biopsy, the histopathological diagnosis made by a pathologist or dermatopathologist will identify the lesion as a benign, atypical or malignant melanocytic proliferation. This determination will dictate whether the region is monitored or additional surgeries are performed. **(C)** Recommendations for additional therapy, such as potential sentinel lymph node biopsy and systemic adjuvant therapy, are made based on the estimated risk of metastasis, occurrence of metastasis, and evidence of treatment failure.

The diversity of clinical circumstances surrounding individual melanomas ensures that there is no “one-size-fits-all” approach to the diagnosis and management of melanoma. Often, the first decision for a clinician in the evaluation of a pigmented skin lesion is to determine whether it is likely benign or has atypical clinical characteristics that warrant biopsy for further evaluation (Figure 1A). If a biopsy is performed, a pathologist must evaluate the likelihood of the lesion being malignant or benign (Figure 1B). For invasive malignant melanomas, a surgical oncologist must decide whether to offer SLNB to evaluate for early metastatic disease (Figure 1C), and selection of systemic therapy becomes necessary with local (e.g., a positive SLNB) or distant metastasis. Each of these decisions represent a junction where the clinician is faced with potential uncertainty and is forced to balance the odds of disease with a significant intervention. Clarification of uncertainty at these decision points might be achieved with objective biomarkers, as discussed in detail below. While these points do not comprise an exhaustive set of decision points in melanoma diagnosis and management that could benefit from biomarker development, tests that aid these decision points would lead to dramatic improvements in patient care. To build a foundation for this discussion, a summary of the fundamental theory of biomarker accuracy and utility is presented.

### The Theoretical Framework of a Clinically Useful Biomarker

A clinically useful test must be capable of changing clinical decision making. In other words, usefulness is determined by the clinician ordering the test. Clinicians routinely encounter patients presenting with an ambiguous disease state. An illustrative example would be a sore throat that could be due to either *Streptococcus pyogenes* or a viral etiology. In this scenario, a rapid “strep” test or bacterial cultures may be ordered to resolve this ambiguity by increasing the clinician's certainty on whether the intervention of antibiotics would benefit the patient. A clinical test could resolve this uncertainty by decreasing the perceived likelihood of the disease state (exclusion), such as with a negative point-of-care rapid “strep” test. Alternatively, a clinical test could increase the likelihood of a disease state (confirmation), such as with a positive bacterial culture. If the test result shifts the clinician's certainty on the patient's diagnosis above or below their self-determined intervention threshold (e.g., a positive rapid “strep” test resulting in an antibiotic prescription), the test will have usefully influenced a clinical decision.

Similarly, predictive and prognostic tests may aim to determine disease trajectory and can assist in directing further intervention, such as follow up imaging or adjuvant therapy selection.

**Figure 2:**
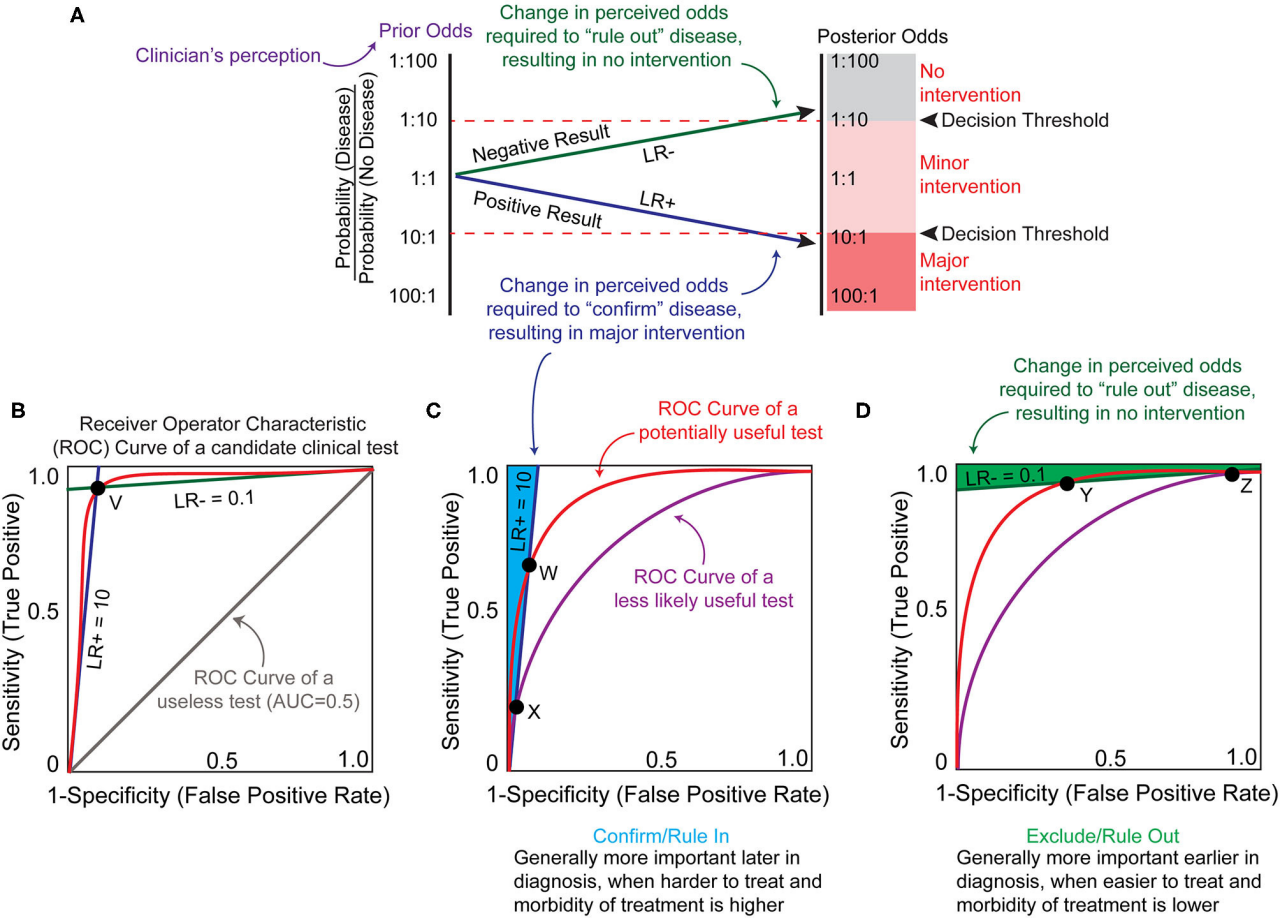
Theoretical framework for biomarker utility analysis. **(A)** A clinician may be challenged with a state of uncertainty, where the perceived probability of the disease (prior odds, purple) does not surpass the clinician's personal thresholds for recommending either major intervention or no intervention (decision thresholds, red dotted lines). A clinician may hope to reduce uncertainty based upon a biomarker test result. A positive result must alter the probability of the disease sufficiently to breach the decision threshold to recommend major intervention (blue line). A negative result must alter the probability of the disease sufficiently to breach the decision threshold to recommend no intervention (green line). In this example, the prior odds are an equal probability of disease or no disease. Based upon this clinician's decision thresholds, an actionable biomarker test result would require either a 10-fold increase or 10-fold decrease in this perceived probability (posterior odds). The extent by which a biomarker test might change the perceived odds can be calculated as a likelihood ratio (LR). A positive LR (LR+, blue line) increases the likelihood of the disease state being present and a negative LR (LR–, green line) decreases the likelihood of the disease state being present. The posterior odds following a biomarker test can be calculated as the prior odds multiplied by the likelihood ratio. **(B)** LR+ and LR– can be plotted against sensitivity and specificity on the axes of a receiver operator characteristic (ROC) curve graph, which represents the sensitivity and specificity of the test at different cut-off values where an “abnormal” result flag would be generated (red curved line). The LR values represent the slope at any given point on the ROC curve, and the LR lines for point V are represented. The area under the ROC curve (AUC) describes the overall sensitivity and specificity of a test at all cut-off points. AUC of 1.0 represents 100% sensitivity and specificity, and a useless test (gray line) has an AUC of 0.5. The red ROC curve has an approximate AUC of >0.9. **(C)** In the example shown in **(A)**, a minimum LR+ of 10 is required to generate posterior odds that breach the clinician's decision threshold for major intervention. The area (shaded in light blue) left of this LR+ slope (dark blue line) represents the sensitivity and specificity characteristics of a test useful for confirming or ruling in a disease state that has an LR+ ≥10. A biomarker test with performance represented by the red ROC curve has a greater range of cut-off values that can produce an LR+ ≥ 10, and has a higher maximal potential sensitivity (point W) compared to the purple ROC curve (point X). This translates to a greater odds of achieving a true test value that has the potential to change the clinician's decision for the red ROC curve, especially with test cut-off values at point W. **(D)** Similar to **(C)**, the area (shaded in green) above the LR– slope (dark green line) represents the sensitivity and specificity characteristics of a test useful for excluding or ruling out the example disease state from **(A)**. The red ROC curve represents a hypothetically useful test with a higher maximal sensitivity capable of achieving a LR– ≤0.1 (point Y), and the purple ROC curve represents a test that is less likely to influence clinical decision making with a lower maximal sensitivity capable of achieving a LR– ≤ 0.1 (point Z). Figure adapted from (23).

The potential utility of a biomarker can be estimated by first considering clinicians' certainty of a disease state using current standard of care (“prior odds”) and the certainty threshold that must be breached for intervention (“threshold”) (Figure 2A). It is important to recognize that since both values are derived from individual clinicians' perceptions, they are inherently imprecise and inconsistent. However, ranges for these values can be estimated through analysis of collective clinical decisions or surveying and these ranges are informative when considering the potential utility of a candidate test. When prior odds and intervention thresholds have been estimated, the slope of the line that connects them represents the change in the odds (also called the likelihood ratio or LR) required to change a decision. A clinical test that decreases the perceived likelihood of the disease state results in a negative likelihood ratio or LR- (Figure 2A, green line). A clinical test that increases the perceived likelihood of the disease state results in a positive likelihood ratio or LR+ (Figure 2A, blue line).

When determining whether a decision point might benefit from biomarker development, it is useful to estimate what values of LR would be required of a theoretical test for posterior odds to surpass the intervention threshold (Figure 2A). Likelihood ratios are calculated from the sensitivity and specificity of the test at one reference range or cut-off value [For information on how to calculate these and other established clinical accuracy metrics see (24) and (23)]. Sensitivity describes the ability of the test to discern the presence of a disease and specificity quantifies how often it correctly identifies disease absence. There is often a trade-off between sensitivity and specificity, particularly with test outcomes reported as a continuous variable. Since clinical accuracy is based on assignment of the test cut-off value (a value outside of the “normal” reference range that is flagged as an abnormal result), the sensitivity and specificity of a test can vary wildly based on where a laboratory assigns the cut-off values for normal. To address this issue, test characteristics can be plotted as a receiver operating characteristic (ROC) curve, which plots the sensitivity and specificity at different cut-off points. This also allows one to compare different tests/biomarkers by calculating the area under the curve (AUC). In general, the higher area under the curve reflects a better performing test, with an AUC of 1.0 having perfect sensitivity and specificity and an AUC of 0.5 being useless (Figure 2B). Since utility is determined by the clinician ordering and interpreting the test, there is no standardized AUC at which a test becomes clinically useful. Instead, positive and negative likelihood ratios can be derived from the slope between any ROC point and the (0,0) and (1,1) vertices, respectively (Figure 2B, point V). If a biomarker test application requires at least a LR+ of 10 and/or LR– of 0.1 to change clinical decision (as in the example presented in Figure 2A), then theoretically any point on the ROC curve that lies to the left (for LR+) or above (LR–) of the LR lines could be considered tests worth further development (Figures 2C,D). However, ROC curves with higher AUC will provide a larger range of potential test cut-offs that will meet these minimal LRs and will consequently have a higher sensitivity and/or specificity at those cut-offs (Figures 2C,D, ROC curves at point W and Y).

In the following sections we apply this theory to estimate the test characteristics required to change clinical decision making in the diagnosis and treatment of melanoma. Where available, we use a combination of published studies and surveys to estimate ranges for the prior odds and intervention thresholds. However, every providers' internal threshold of certainty for clinical decision making will vary in the context of patient-specific factors in addition to test characteristics. Thus, these ranges should not be considered exact quantifications but rather estimates to help determine which biomarkers may be worth investing community resources for further discovery and development.

### Toward Enhancing Pigmented Lesion Screening With Biomarkers

Many factors are considered when assessing a pigmented lesion in clinic prior to recommending a biopsy. These include but are not limited to clinical impression of the lesion, history of the lesion (either as reported by the patient or through comparison to previous clinical images), the patient's history and risk for melanoma, total number of lesions, and dermatoscopic features. Since the decision to biopsy represents a gestalt of these different factors, it is not surprising that the level of specialty training and experience of the physician substantially alter the number of lesions biopsied for each melanoma identified. A recent meta-analysis of 351 studies determined the sensitivity and specificity of identifying melanomas during skin exams was 87.5 and 81.4%, respectively, for dermatologists, but only 79.9 and 70.9% for primary care physicians (25). Unfortunately, access to dermatologists for regular skin exams is neither uniform nor comprehensive, with over half of U.S. citizens having insufficient access to dermatologic care (26). Dermoscopy and reflectance confocal microscopy (RCM) are two non-invasive approaches that increase the number of melanomas biopsied, while reducing the number of benign lesions biopsied (27, 28). However, these techniques also require highly skilled interpretation, and use of RCM is largely limited to dermatologists who specialize in pigmented lesions. Dermoscopy is more broadly used in clinical practice, though it does not appear to increase diagnostic accuracy in the practice of non-experts (29) and has even been reported to reduce sensitivity of melanoma detection compared to clinical assessment alone (30). An objective biomarker could therefore improve the odds to biopsy a true melanoma, especially in situations where a specialized dermatologist and auxiliary equipment/techniques are not available. In contrast, any increases in certainty might be minimal when used by a highly specialized and experienced pigmented lesion dermatologist.

**Figure 3:**
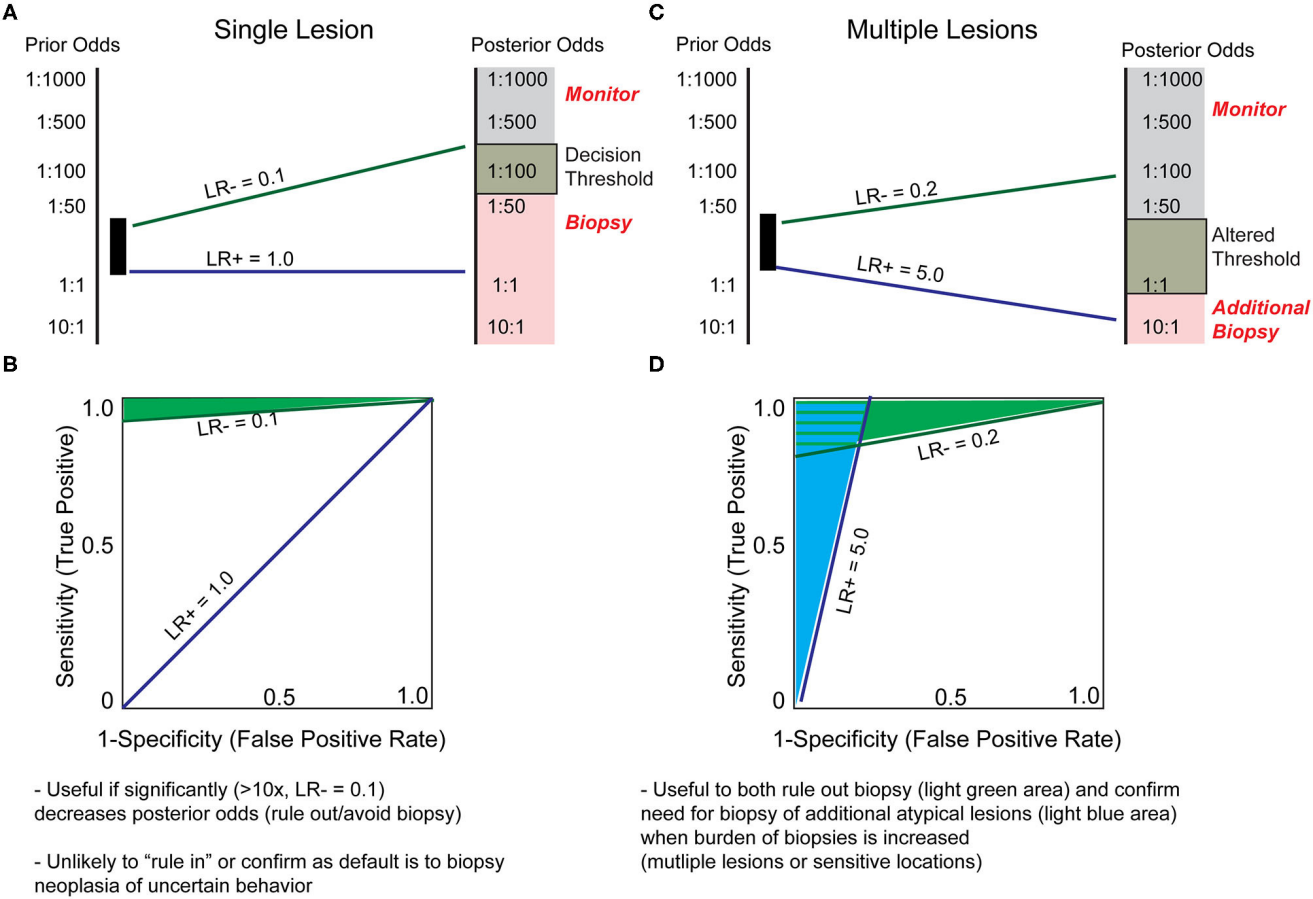
Hypothetical biomarker test characteristics to assist in deciding to biopsy. **(A)** Solitary lesions with perceived prior odds of malignancy estimated at 1:2 to 1:30 are typically biopsied due to the minimal pain and expense of biopsy. Therefore, a test's LR+ characteristic is not generally valuable when evaluating a single lesion. In contrast, a LR– of 0.1 indicates a 10-fold decrease in likelihood of malignancy, and may inform the decision to not biopsy. **(B)** LR+ and LR- slopes from **(A)** plotted on a receiver operating characteristic (ROC) curve, indicate the hypothetical test characteristics required of a biomarker to provide clinically meaningful decision to biopsy a suspicious lesion, with the area shaded in green representing estimated test characteristics useful in ruling out biopsy. **(C)** The estimations and assumptions change when a single patient presents with numerous borderline lesions. Due to limited patient tolerance for multiple biopsies, there may be an increased threshold to biopsy each additional lesion. A test designed to address these parameters would have different test characteristics. For instance, a LR– ≤ 0.2 might convince a provider to defer biopsy on a lesion, and a LR+ ≥5 could prompt a biopsy for a lesion that would have otherwise been monitored. **(D)** In the setting of multiple lesions, the question of which lesions to biopsy may be more readily influenced with biomarker testing, as indicated by the light blue shaded area (shift from indecision to biopsy) or light green shaded area (shift from indecision to monitor). These assumptions representing a higher burden of biopsy and altered threshold may also apply to biopsies of sensitive areas such as face, genitals, or nail bed.

Ultimately, the cost of a skin biopsy to the individual patient, in terms of both morbidity and finance, is relatively low, but the cost of leaving an early-stage melanoma undiagnosed is exceptionally high. By applying the above theory of biomarker utility, the prior odds of malignancy can be estimated by considering the number of lesions biopsied to diagnose one melanoma. This ranges from ~2 for some dermatologists to ~30 for primary care physicians (31). Thus, according to current clinical practices, any lesions with prior odds lower than 1/2–1/30 of being malignant are less likely to be biopsied. A biomarker introduced at this stage could therefore provide utility if it conferred a significant degree of certainty that a lesion was not malignant and thus avoided biopsy. For certainty to be sufficiently increased such that the perceived odds of malignancy is reduced from 1/2 and 1/30 prior to the test to <1/30 after the test (posterior odds), an estimated likelihood ratio of 0.1 (LR-) is required (Figures 3A,B, green line). Conversely, for a melanocytic tumor eliciting a high suspicion of malignancy from conventional means (high prior odds), a biomarker is unlikely to change decision making, as the clinical threshold to perform the outcome (biopsy) has already been met (LR+ 1.0, Figures 3A,B, blue line). Although this analysis suggests that molecular biomarkers are unlikely to change clinical behavior when assessing solitary lesions, there are other clinical scenarios that challenge the assumptions and conclusions of this model. For example, when individual lesions present in sensitive areas, such as the face or genitals, the morbidity of biopsy and the clinical suspicion threshold required to biopsy are increased. In these cases, a modest LR– may suffice to shift the clinical decision from “biopsy” to “monitor.” Alternatively, due to increased morbidity and decreased patient tolerance of multiple skin biopsies, a patient with many suspicious lesions may benefit from a combination “rule in” and “rule out” molecular test (necessary characteristics estimated as LR+ = 5.0 and LR– 0.2, respectively, Figures 3C,D) to prioritize lesions to biopsy (32).

The pigmented lesion assay (PLA), developed by DermTech, is the only biomarker currently commercially available for clinical use addressing this decision (33, 34). This test is an adhesive patch-based gene expression test measuring expression of *PRAME* and *LINC00518*, with reported sensitivity of 91% and specificity of 69% (34). The non-invasive nature and high sensitivity of this test has allowed for clinical incorporation in certain specialized scenarios (32). However, a recent study concluded the test is neither widely nor routinely used by pigmented lesion experts, which is consistent with predictions of the above model (35). Beyond the test's performance characteristics, another limitation is the delay between sampling and result, which necessitates a subsequent biopsy visit if the test is positive. If a similarly sensitive point-of-care biomarker test could be developed as a rapid in-office test, it may be more widely adopted.

In summary, because the clinical default in a setting of uncertainty will usually be to biopsy an indeterminate lesion, biomarkers with a large LR– represent the opportunity to change clinical management by avoiding biopsy. For this purpose, a biomarker with maximum specificity, thus minimizing false negatives, is critical since the adverse consequences of missing the diagnosis is high and the morbidity and cost of a skin biopsy is relatively low. There are situations within a dermatology clinic where a biomarker with high sensitivity (LR+) may also prove clinically useful, but these are likely to be more specialized scenarios such as prioritizing lesions for biopsy in a patient with dysplastic nevus syndrome. Combined with practical limitations for such widespread use of non-invasive biomarkers used at this stage, including the ease of use, cost, and the timing of results, we speculate that current technologies for molecular profiling are unlikely to be influential in the routine evaluation of single lesions.

### Current Biomarkers for the Histopathological Diagnosis of Pigmented Lesions

**Figure 4:**
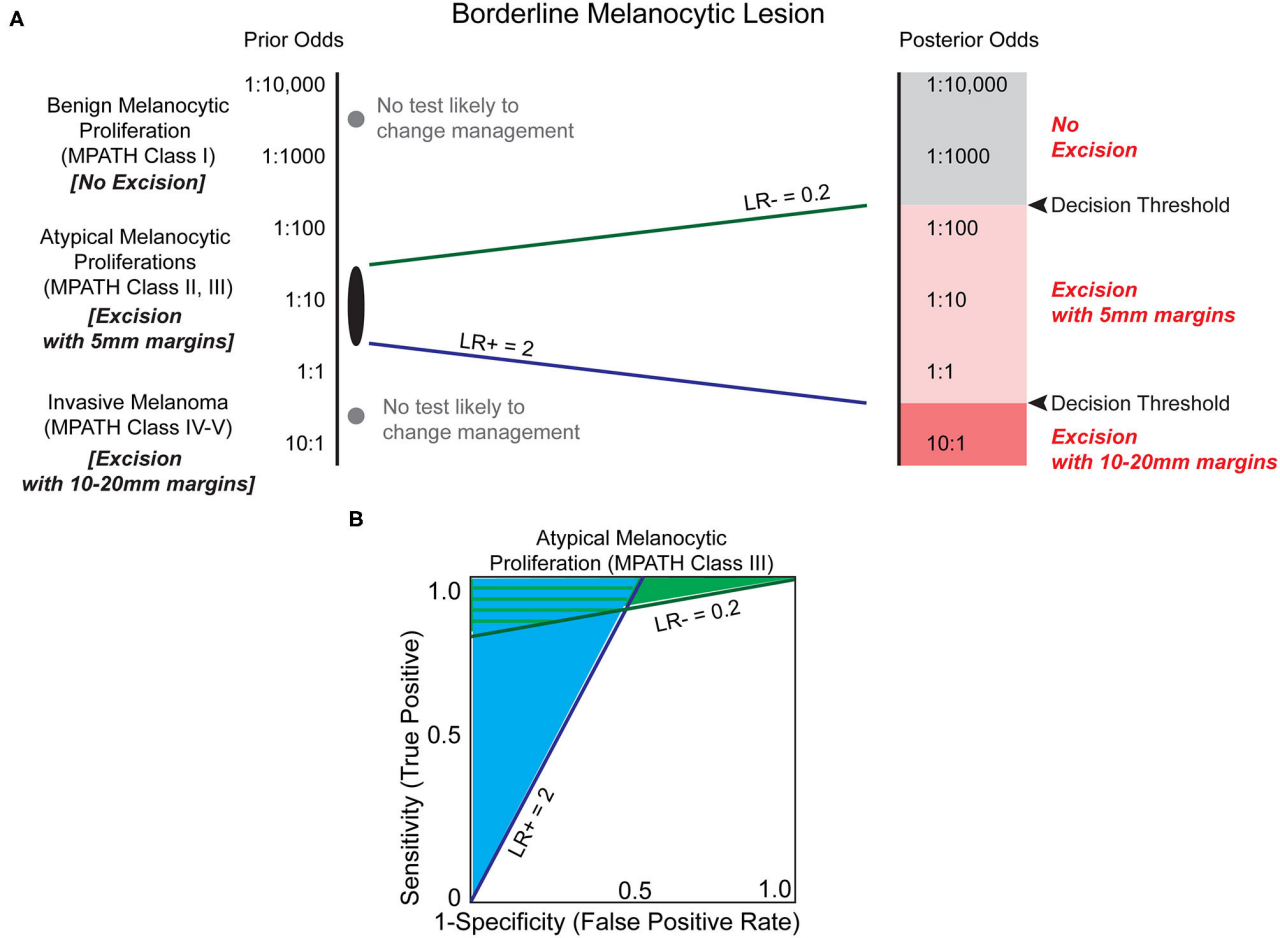
Biomarkers have the potential to clarify the risk of melanoma in the histopathologic interpretation of borderline pigmented lesions. **(A)** Melanocytic lesions exist on a histologic spectrum from benign-appearing to malignant. At the extremes of this spectrum (e.g., unambiguous benign melanocytic nevus (MPATH-Dx class I) or unambiguous invasive melanoma (MPATH-Dx class IV-V) no biomarker test is likely to change management (gray dots, Table 1). For borderline melanocytic lesions, especially MPATH-Dx class II-III lesions, pathologists are generally conservative and recommend excision of ambiguous lesions that may have no malignant potential to avoid potential adverse consequences of underdiagnosis (36). This results in excision using 5 mm margins of many atypical and dysplastic melanocytic lesions without full knowledge of malignant potential. A LR– of 0.2, corresponding to a 5-fold reduction in estimated malignant potential, may be sufficient to avoid a recommendation of excision in a more benign-appearing atypical/dysplastic specimen if threshold for treatment is set at ~1:200. At the more dysplastic end of the spectrum, a biomarker test with LR+ of 2 may be sufficient to increase recommendation from excision with 5 mm margins to excision with 10 mm margins. This results in a significant increase in size of total excision and repair that is commensurate with the higher estimated increased risk of malignancy. Due to the inherently ambiguous nature of MPATH-Dx class II–III lesions, it is challenging to estimate the decision thresholds for treatment. Depending on the pathologist's level of suspicion and the clinician's threshold for intervention (which may be influenced by patient specific-factors including prior odds and morbidity of procedure), different LR values are almost certainly necessary to justify changing intervention, such as deferring excision or increasing excision margins. **(B)** The estimated minimum necessary likelihood ratios indicate that there is a significant range of biomarker characteristics that provide clinical utility in deferring excision (light green shaded area) and/or increasing excision margins (light blue shaded area).

The established challenges of histopathologic analysis has motivated the search for biomarkers to assist in melanoma diagnosis. If prior odds based on original histology favor a more benign dysplasia, such as a dysplastic nevus, then it is possible that a moderate LR–, estimated at 0.2, may convince a clinician to monitor a biopsy site rather than pursuing an excision with margins (Figure 4). Conversely, if a biomarker applied to the same lesion demonstrated a significant LR+ of 10, then the result may convince a clinician to excise the area with larger margins (10–20 mm instead of 5 mm), despite relatively low prior odds. For a lesion with higher prior odds of possessing malignant potential but lacking *bona fide* features of melanoma, often termed an atypical melanocytic proliferation, a less compelling LR+ of 2 might be sufficient to upgrade a diagnosis to melanoma, whereas a significantly lower LR- of 0.1–0.01 might be required to decrease excision margins or avoid excision altogether. These analyses demonstrate that there could be great clinical utility of biomarkers examining the malignant potential of intermediate melanocytic lesions, particularly when the biomarker holds a significant likelihood ratio that modifies the prior odds by an order of magnitude or more.

Numerous biomarker tests have been developed to address this issue. Table 2 reports biomarker tests that are currently commercially available or used in selected referral centers and Table 3 (discussed below) reports additional candidate biomarker tests reported in the literature. Immunohistochemistry (IHC) biomarkers are usually the most easily adopted test with regards to tissue requirement, cost, and required laboratory equipment and expertise. One promising IHC marker is PRAME (Preferentially expressed Antigen in Melanoma), which has demonstrated an LR+ of 29–62.5 and an LR- of 0.13–0.253 when assessed with an independent validation cohort (39, 40). Considering that it shares 90% concordance with fluorescent *in situ* hybridization (FISH) and 92.7% agreement with the final diagnostic interpretation (40), adopting PRAME IHC could be an economical option for most laboratories to use for routine differentiation of nevi and melanoma. Comparatively, p16 IHC, which is used at some institutions to help distinguish between nevi and melanoma in challenging cases, has no published validation cohorts to identify the clinical characteristics of this test. As a result, the accuracy, reliability, and versatility of p16 is unclear. In addition, the utility of p16 expression depends on the type of lesion since it may help distinguish desmoplastic Spitz nevi from desmoplastic melanoma (37), but fails at distinguishing Spitz nevi from Spitzoid melanoma (38).

**Table 2:** Melanoma biomarkers used by reference centers and dermatopathologists.

**Category**	**Method class**	**Method specifics**	**Total study number**	**Study breakdown**	**Estimated tissue requirement**	**Study objective**	**Number of pathologists confirming cohort**	**AUC**	**S/Sp/PPV/NPV, LR+/LR–^**‡**^**	Independent cohorts assessed^**^	**Qualitative vs. quantitative**	**Citation**
**Individual biomarker assays**												
Protein expression	IHC	Loss of p16 protein expression	26	11 desmoplastic melanomas, 15 Spitz nevi	Sectioned FFPE slide	Differentiate desmoplastic Spitz nevus and desmoplastic melanoma	NR	NR	82^*^/100^*^/NR/NR, >80/0.18	No	Qualitative, requires expert interpretation	(37)
			37	19 Spitzoid melanomas, 18 Spitz nevi	Sectioned FFPE slide	Does not differentiate Spitzoid melanomas from Spitz nevi	Initial diagnosis plus consensus conference with 3+ dermatopathologists	NR	21^*^/83^*^/NR/NR, 1.2/0.95			(38)
		PRAME protein expression (4+ staining)	400	255 primary and metastatic melanomas, 145 melanocytic nevi	Sectioned FFPE slide	Support diagnosis of melanoma by PRAME positivity	2+	NR	87^*^/97^*^/NR/NR, 29/0.13	Yes	Qualitative, requires expert interpretation	(39)
			110	110 ambiguous cases reviewed by 2+ dermato-pathologists	Sectioned FFPE slide	Validate and compare PRAME IHC to FISH for differentiating ambiguous cases	2+	NR	75.0/98.8/NR/NR, >62.5, 0.253			(40)
Copy Number Variation	CGH	Copy number variation by comparative genomic hybridization	186	132 melanomas, 54 benign nevi (27 Spitz nevi, 19 blue nevi, 7 congenital nevi)	High tissue requirement; remainder of FFPE block	Differentiate melanoma from benign nevi on basis of copy number variation	NR	NR	96^*^/87^*^/NR/NR, 7.4/0.046	No	Qualitative, requires expert interpretation	(41)
FISH	FISH	Four probes targeting chromosome 6p23, 6p25, 11q13, and centromere 6	22	12 ambiguous lesions, 10 unequivocal lesions	Medium to High tissue requirement	Validate FISH on histologically ambiguous lesions based on clinical behavior of lesion	Initial diagnosis plus one dermatopathologist review, ambiguous tumors reviewed by two pathologists	NR	60/50/NR/NR, 1.2/0.80	Yes	Qualitative, requires expert interpretation	(42)
		Four probes targeting chromosome 9p21, 6p25, 11q13, and 8q24	424	Training set: 152 melanoma and 170 nevi. Validation set: 51 melanoma and 51 nevi.	Medium to High tissue requirement	Distinguish between melanoma and nevi on basis of chromosomal changes	1	0.94+	94/98/NR/NR, 47/0.061	Yes		(43)
Gene Expression with Myriad myPath	23 GEP qRT-PCR	Weighted 23 gene expression algorithm by qRT-PCR	901	Training set: 254 melanoma and 210 nevi. Validation set: 437 independent lesions	Medium tissue requirement, macro-dissection from sections	Differentiate benign nevi from malignant melanoma	Initial diagnosis, one study dermatopathologist review, if discordance a third dermatopathologist	0.96	90/91/NR/NR, 10/0.11	Yes	Quantitative algorithm, Qualitative output	(44)
			1,400	204 melanoma, 656 nevi		Prospective validation of differentiating between benign and malignant melanocytic lesions	Concordance between 3 experienced dermatopathologists	NR	91.5/92/NR/NR, 11.4/0.0924			(45)
			182	99 melanomas, 83 nevi		Correlate long-term clinical outcomes with gene signature	3	NR	93.8/96.2/NR/NR, 24.7/0.0644			(46)
			50	23 melanomas, 27 nevi		Categorize potential desmoplastic melanomas as likely malignant or likely benign	2 dermatopathologists, independent review	NR	Sensitivity is “about 80%, better than FISH”			(47)
			181	125 diagnostically uncertain lesions, 56 diagnostically certain lesions		Test accuracy of GEP to diagnosis and outcomes (gold standard) in cases with uncertain histo-pathological diagnoses	3 dermatopathologists	NR	90.4/95.5/NR/NR, 20.1/0.101			(48)
**Compared techniques**	**Total study number**	**Study breakdown**	**“Gold Standard”**	**Number of pathologists confirming cohort**		**Unequivocal histology: S/Sp/PPV/NPV, LR+/LR–^‡^**	**Unequivocal Concordance**	**Challenging histology: S/Sp/PPV/NPV, LR+/LR–^‡^**		**Challenging Concordance**	**Citation**	
**Comparison of biomarker assays**												
CGH vs. FISH	30	25 melanomas, 5 nevi	Histology	Confirmation of initial diagnosis by 1 dermatopathologist		–	FISH vs. CGH: 90%	–		–	(49)	
Histology vs. FISH vs. myPath	117	Histologically unequivocal: 15 malignant and 24 benign. 78 Histologically challenging cases	Histology	1 pathologist for unequivocal and 3 for challenging cases		FISH: 93/100/NR/NR, >90/0.07 myPath: 62/95/NR/NR, 12.4/0.40	FISH vs. histology: 97%, myPath vs. histology: 83% FISH vs. myPath: 80%	FISH: 56/83/NR/NR, 3.3/0.53 myPath: 52/80/NR/NR, 2.6/0.60		FISH vs. histology: 70%, myPath vs. histology: 64% FISH vs. myPath: 70%	(50)	
Histology vs. myPath vs. FISH vs. (discordant cases only) CGH	268	Histologically unequivocal: 198. Histologically challenging: 70	Histology and SNP-array	Challenging cases reviewed by 1 study and 2 independent dermatopathologists		myPath: 67/81/NR/NR, 3.5/0.41	myPath vs. histology: 75%	FISH: 61/100/NR/NR, >60/0.41 myPath: 50/93/NR/NR, 7.1/0.53		FISH vs. histology: 84%, myPATH vs. histology: 74% FISH vs. myPath: 69%Agreement with nine pathologists in discordant cases: CGH vs. histology: 71% FISH vs. histology: 54% MyPath vs. histology: 14%	(51)	

AUC, area under (receiver operating characteristic) curve; CGH, comparative genomic hybridization; FFPE, formalin-fixed paraffin embedded; FISH, Fluorescence in situ hybridization; IHC, immunohistochemistry; LR+, positive likelihood radio; LR–, negative likelihood radio; NPV, negative predictive value; NR, not reported; PPV, positive predictive value; qRT-PCR, quantitative real time polymerase chain reaction; S, sensitivity; Sp, specificity.

* Calculated from manuscript data, no validation cohort.

** Tests are considered to have independent cohort validation if an independent clinical cohort was included in the original study or at least one follow-up study used an independent cohort with the same diagnoses or a cohort with ambiguous diagnoses.

‡ *All LR values are calculated based on reported sensitivity and specificity*.

**Table 3:** Proposed melanoma diagnosis biomarkers and prospective uses.

**Category**	**Method class**	**Method specifics**	**Total study number**	**Study breakdown**	**Estimated tissue requirement**	**Study objective**	**Number of pathologists confirming cohort**	**AUC**	**S/Sp/PPV/NPV (%), LR+/LR-^**‡**^**	**Qualitative vs. quantitative**	**Citation**
DNA Methylation	40-CpG classifier	Next generation sequencing	162	89 melanoma, 73 nevi	250–300 ng of DNA from microdissected slides	Classify uncertain samples as melanoma or nevi	4	0.996	96.6/100/ 100/96.2, >90/0.03	Quantitative	(52)
	Promoter methylation	1,505 CpG site microarray, analyzed 12 CpG loci highly predictive of melanoma	49	22 melanoma, 27 nevi	250 ng of DNA from FFPE tissue	Differentiate nevi and malignant melanoma	NR	0.89–1.0	≤ 100/ ≤ 100/NR/NR	Quantitative	(53)
		CpG island hypermethylation in promoter of *CLDN11*	405	199 melanoma, 208 nevi	Not reported, extraction from tissue	Differentiate dysplastic nevi and malignant melanoma	NR	0.806	52/94/ /NR/NR, 8.67/0.51	Quantitative	(54)
		*RASSF1A* promoter methylation in serum cell-Free DNA	152	84 melanoma, 68 nevi	Serum draw	Differentiate healthy vs. *in-situ*, invasive, and metastatic melanoma	NR	0.905	NR	Quantitative	(55)
Gene expression	microRNA ratio	Sequencing or qRT-PCR to obtain microRNA Ratio Trained Model score	179	106 melanoma, 73 nevi	Single tissue section	Differentiate nevi and malignant melanoma	5+	0.9	81/88/ NR/NR, 6.75/0.22	Quantitative	(56)
	RT-PCR	qRT-PCR for *SILV*	193	47 melanoma, 48 nevi	3–12 tissue sections	Differentiate dysplastic nevi and malignant melanoma	NR	0.94	NR	Quantitative	(57)
	Microarray	DNA microarray assay using 14 genes	120	62 melanoma, 58 nevi	8 μM tissue section, microdissected with laser capture	Differentiate nevi and malignant melanoma	2–4	NR	90/86/NR/ NR^*^, 6.43/0.10	Quantitative	(58)
Serum nucleic acid	Circulating miRNA panel	qRT-PCR for six microRNAs in serum	65	Serum from 50 uveal melanoma, 5 metastatic uveal melanoma, 10 uveal nevi	Serum draw	Differentiate uveal melanoma from nevi	NR	NR for panel	93/100^*^/100/76, 20/0.07	Quantitative	(59)
Protein Expression	Ciliation index	Immunofluorescence for acetylated alpha-Tubulin	124	26 melanoma, 42 nevi	Single tissue section	Classification of Spitzoid tumors as malignant or benign	3+	0.84	81/65/NR/NR, 2.31/0.29	Semi- quantitative	(60)
	IHC	IHC for 5-hydroxymethyl-cystosine	190	126 melanoma, 45 nevi	Single tissue section	Differentiate nevi from melanoma	2	0.78	93/98/NR/NR, 46.5/0.07	Semi-quantitative	(61)
	Multi-IHC semi-quantitative scoring system	IHC scoring of Ki-67, p16, and HMB45	308	234 melanoma, 74 nevi	Three tissue sections	Differentiate nevi and malignant melanoma	NR	0.987	97.4/97.3/NR/NR, 36.1/0.03	Semi-quantitative	(62)

AUC, area under (receiver operating characteristic) curve; FFPE, formalin-fixed paraffin embedded; LR+, positive likelihood radio; LR–, negative likelihood radio; NPV, negative predictive value; NR, not reported; PPV, positive predictive value; S, sensitivity; Sp, specificity; qRT-PCR, quantitative real time polymerase chain reaction.

* Leave 1-out validation.

‡ *All LR values are calculated based on reported sensitivity and specificity*.

FISH is another tissue staining modality that has been used for over a decade to help distinguish nevi from melanoma. It has well-developed scoring criteria, several validation studies, has undergone quality improvement to reduce the number of erroneous Spitzoid classifications, and has been directly compared to both the myPath gene expression panel (GEP) and copy number variation (CNV) assay by comparative genome hybridization (CGH) (42, 43, 49–51). The clinical characteristics of FISH in melanoma diagnosis are highly competitive and cost-effective when compared to the other molecular techniques (Table 2) (43, 49–51). However, evaluating FISH staining requires expert interpretation and expensive microscopy equipment, which limits the adoption of the technique outside of reference laboratories.

The CNV by CGH assay has been used clinically for almost two decades and assesses large genomic mutations and deletions across the genome. Melanomas usually possess multiple chromosomal losses and gains (96.2% frequency), Spitzoid nevi frequently have a unique 11p amplification, and non-Spitzoid nevi usually have no detectable chromosomal changes (41). While a powerful technique to distinguish nevi from melanomas, there is no clear CNV-based definition of melanoma. As a result, the CNV cut-off that separates a benign vs. malignant lesion is often a gestalt decision from the dermatopathologists, which makes quantifying the clinical accuracy characteristics challenging. However, the price of CGH has fallen in recent years, the technology can be readily adopted by laboratories with experience in molecular diagnostics, and, it has 90% concordance with FISH in cases with unequivocal histopathology (49). In particularly challenging cases, CGH also outperformed both FISH and myPath in reaching concordance with a consensus histopathological diagnosis (51).

More recently, the Myriad myPath quantitative gene expression panel (GEP) and scoring algorithm was reported to have favorable test characteristics (AUC of 0.96, sensitivity of 90% and specificity of 91%) (44). This platform has subsequently been studied across prospectively submitted samples (45) in the evaluation of desmoplastic melanocytic proliferations (47) and in conjunction with long-term clinical outcomes (46). The most recent study is well-designed to assess function in histopathologically uncertain lesions and exemplifies how to use clinical outcome data as the gold standard instead of histopathology (48). Despite its impressive clinical characteristics, when assessed individually, the myPath methodology is not as competitive in comparison studies to FISH and CGH when histopathology is the gold standard (50, 51). Additional studies of the myPath test compared directly against FISH and CGH with long term follow up of clinical outcomes as the gold standard are warranted to determine the true relative utility of these tests.

In summary, the existing literature most highly supports the use of PRAME IHC, FISH, and CGH to aid dermatopathologists in routinely stratifying melanoma risk. While the clinical characteristics of CGH are uncertain, the presence of amplifications and deletions would likely prompt a conservative practitioner to upgrade the diagnosis to melanoma (presumed LR+ >2). FISH and PRAME IHC easily reach the modeled threshold of utility, even in challenging cases, but would not be sufficient to rule out melanoma (LR– is not <0.1–0.01) (50, 51, 63), and it is unclear if a CGH result revealing no amplifications or deletions would have a sufficient LR- to overturn a conservative melanoma diagnosis. The only available biomarker that could potentially have such a robust LR– is myPath, but such performance would need to be proven in a head-to-head comparison with the other testing modalities. As it stands, there is no currently used biomarker with a robust enough LR+ and LR– to be definitive for including or excluding a melanoma diagnosis with certainty, which leaves room for the further development of novel tests and more rigorous validation of current tests.

### Potential Future Biomarkers for the Diagnosis of Pigmented Lesions

Since distinguishing early invasive melanoma from pre-malignant melanocytic lesions by routine histopathology remains a diagnostic challenge (15), numerous research groups have attempted to develop ancillary tests to augment melanoma diagnosis. Presented below and summarized in Table 3 are a set of studies that examined promising biomarkers. All of these studies utilize methodologies obtainable by academic reference laboratories, all have at least one test characteristic defined, and most have been assessed in at least one independent test cohort.

One study used IHC to develop a semi-quantitative scoring system based on the percent expression of Ki-67 (MIB-1), p16, and HMB45 expression in melanocytes. Of these commonly available stains, Ki-67 has the best individual sensitivity and specificity, but the addition of p16 and HMB45 increased this to 97.4% sensitivity and 97.3% specificity with an AUC of 0.987. Notably, the IHC panel accurately predicted the metastatic evolution of tumors that were initially classified as melanocytic or Spitzoid nevi, and it appropriately downgraded one melanoma to a benign nevus (62). While these results are impressive, this test requires stain scoring which is notoriously subjective. Therefore, maintaining a high interobserver concordance with three IHC stains could be challenging, especially with scoring borderline p16 and HMB45 cases. Another proposed diagnostic marker is 5-hydroxymethylcytosine (5-hmC), which is decreased in melanoma compared to nevi, keratinocytes, and lymphocytes. Staining variation limits the sensitivity and specificity of 5-hmC scoring system to 92.7 and 97.7%, respectively, though there is a characteristic biphasic staining pattern between the melanoma and nevi components of a transformed nevus that could be clinically useful according to a pilot study (61).

Melanomas lose primary cilia after transformation whereas they are retained in benign nevi (64–66). The presence of primary cilia have been shown to differentiate typical and atypical Spitzoid nevi from malignant tumors with a sensitivity of 81% and specificity of 65% (AUC 0.84), but, with the incorporation of cytologic atypia, hyperchromatism, and asymmetry in making a diagnosis, the combined specificity and AUC were increased (sensitivity 72%, specificity 89%, AUC 0.92) (60). This is an improvement over standard diagnosis of Spitzoid tumors, which are classically difficult to distinguish between benign and malignant subtypes. Recent pilot studies have also suggested visualization of primary cilia using IHC may also provide similar utility in other melanoma types (67–69).

Discrimination between nevus and melanoma may also be accomplished using differences in gene expression. The most accessible method of analysis is to perform quantitative real time polymerase chain reaction (qRT-PCR) on a single transcript target from tissue or serum. For instance, silver homolog (also called *SILV, PMEL, gp100*, or *ME20M*) expression from FFPE tissue is significantly lower in melanomas compared to nevi, and it has favorable characteristics (AUC 0.94) as a single biomarker (57). Similarly, a qRT-PCR panel for 6 microRNAs (miRNA) in serum demonstrated positive and negative likelihood ratios of 20 and 0.07, respectively, in distinguishing uveal melanoma and nevi (59). Additional work remains to be completed to confirm clinical utility of these studies as neither contained validation cohorts and both had small sample sizes. The importance of a validation cohort is highlighted by our 2019 study where we developed a miRNA ratio trained model from miRNAs enriched in microdissected melanoma cells compared to nevus cells. While the initial training model had an AUC of 0.98, the clinical accuracy of the qRT-PCR validation cohort containing whole FFPE sections dropped to a sensitivity of 81%, specificity of 88%, and AUC of 0.90 (56). Likewise, a microarray study on microdissected FFPE tissue found that the expression profiles of 36 genes could be used to identify melanoma with sensitivity (90%) and specificity (88%) in the validation cohort (58). While the gene expression panels described above have test characteristics that are theoretically compatible with a useful clinical test, their reliability is uncertain until larger studies confirm clinical utility.

Of the different classes of proposed biomarkers, DNA methylation testing is not widely available outside of reference laboratory testing for X chromosome disorders and specific genes, such as *MLH1* and *MGMT*. In melanoma, promoter methylation status of two genes have been examined as potential biomarkers. The Ras associated domain family 1 isoform A (*RASSF1A*) gene is a tumor suppressor found to be methylated in melanomas compared to healthy subjects (AUC of 0.905) by cell-free DNA (cfDNA) analysis (55). While cfDNA allows for a non-invasive blood draw and detects *in situ* melanomas with an AUC of 0.945, no studies have been published validating the *RASSF1A* marker. In comparison, the promoter for *CLDN11*, a component of the tight junction, undergoes CpG island methylation in approximately 50% of primary melanomas compared to 3–6% of dysplastic nevi. *CLDN11* promoter methylation status was determined by extraction from FFPE tissue and methylation-specific PCR in a large cohort of melanomas and nevi. This yielded a 52% sensitivity and 94% specificity (AUC of 0.806), suggesting utility for ruling in melanoma in the context of a suspicious dysplastic nevus. Another group developed a CpG site microarray panel and, ultimately, a 40-CpG next generation sequencing (NGS) panel to assess the methylation of status of multiple genes from microdissected FFPE slides. While both of these studies had characteristics compatible with a useful clinical test (sensitivity up to 96.6%, specificity up to 100%, AUC up to 0.996), they are limited by small cohort sizes and lack of long-term patient outcome data (52, 53).

Overall, there are several potential biomarkers that could be used to distinguish early melanoma from dysplastic nevi. All of the above tests have LRs (Table 3) that could be beneficial in distinguishing atypical melanocytic proliferations from melanoma based on our model (Figure 4). Of these biomarkers, we speculate that the most accessible, least complex, minimally invasive, and relatively affordable test that could be readily adopted into the laboratory is 5-hmC IHC (61). Prior to its adoption, however, large cohort studies are required to confirm the promising initial results. Of all of the tests included in Tables 2, 3, the 40-CpG classifier next generation sequencing test for DNA methylation has the best overall combined characteristics (52). If future multi-institutional studies show good concordance and validation of the test characteristics, the 40-CpG classifier test could be useful for assessing the most challenging cases of atypical melanocytic proliferations. However, the major limitation of this test is the NGS approach: ample tumor tissue will be required, the method is expensive, and turn-around-time will likely be at least 8–14 days. Thus, other tests that require less tissue, cost, time, and expert interpretation, such as the multi-IHC panel or ciliation index, also warrant further consideration (62, 68).

### Potential Biomarkers for Staging, Prognosis, and Treatment Effect Monitoring

In addition to indications for biopsy and diagnostic accuracy, potential biomarkers were assessed for other important clinical decision points related to the staging and treatment of invasive melanomas (Figure 1C, Table 4).

**Table 4:** Proposed prognostic, staging, and treatment-monitoring biomarkers.

**Category**	**Method class**	**Method specifics**	**Total study number**	**Study breakdown**	**Estimated tissue requirement**	**Study objective**	**Number of pathologists confirming cohort**	**AUC**	**S/Sp/PPV/NPV (%), LR+/LR-^**‡**^**	**Qualitative vs. quantitative**	**Citation**
**Prediction of sentinel lymph node biopsy status**											
Castle DecisionDx	31 GEP	TaqMan qRT-PCR from FFPE tissue	1421	By T: 613 T1, 452 T2, 240 T3, 116 T4	macrodissected FFPE sections	Identify T1–T2 melanomas at low risk for positive SLNB	NR	NR	NR	Quantitative	(70)
Skyline	GEP and clinico-pathologic features	TaqMan qRT-PCR from FFPE tissue	506	160 discovery, 360 development, 146 validation	FFPE blocks	Develop and validate a GEP panel to identify positive SLNB	2+ dermato-pathologists	0.78	89/76/NR/NR, 3.71/0.14	Quantitative GEP, qualitative clinicopathologic features	(71)
			754	128 positive SLNB, 626 negative SLNB	FFPE blocks	Identify patients who can forgo SLNB	Initial diagnosis made with 2+ dermato-pathologists. No other review	0.82	TIb: 41/82/7/98, 2.28/0.72; TIIa: 80/53/21/95, 1.7/0.24; TIIb: 94/27/21/96, 1.29/0.22; TIIIa: 99/12/33/98, 1.13/0.08; TIIIb: 100/7/45/100, 1.08/ <0.01		(72)
**Metastasis identification**											
Circulating Tumor Cells	Flow and fluorescent microscopy	CD146 cell sorting. DAPI+, HMW-MAA+, CD45/CD34– Immuno-fluorescence	15	Tumor: 15, 0. Metastasis 6, 9	Whole blood	Identification of early mets	NR	NR	CTC: 33/100/100/69, >30/0.67; CTC+5-S-cysteinyldopa: 67/100/100/82, >60/0.33	Semi-quantitative	(73)
Serum nucleic acid	Circulating non-coding RNA by qRT-PCR	hsa-miR-1246 miScript assay for RNU2-1f	122	33 training, 16 distant metastasis validation, 16 nevi, 57 healthy control	Serum draw	Use RNU2-1f non-coding RNA serum level to diagnose melanoma mets	NR	0.9375 (validation)	Regional mets: 69.6/87.2/NR/NR Distant mets: 70.0/87.2/NR/NR	Quantitative	(74)
Protein Expression	ELISA	ELISA for S100 and OPN	106	6 squamous cell carcinomas, 76 melanomas, 24 metastatic mela-nomas, 3 normal	Plasma draw	Distinguish metastatic melanoma from local disease with OPN +/– S100 and LDH	NR	OPN and S100: 0.97	95.5/85.9/NR/NR, 6.77/0.05	Quantitative	(75)
**Prognosis**											
Castle DecisionDx	31 GEP	TaqMan qRT-PCR from FFPE tissue	479	107 discovery, 268 development, 104 validation	macrodissected FFPE sections	Identification of stage I/II tumors with worse prognosis	NR	0.93	100/78/NR/NR, 4.54/ <0.01	Quantitative	(76)
			217	All post-SLNB: 58 positive SLN, 159 negative SLN	macrodissected FFPE sections	Prognostic accuracy of GEP and SLNB in predicting RFS, distant mets, and OS	NR	NR	N.R./N.R./50/82		(77)
			205	By stage: 2 I, 68 IA, 39 IB, 1 II, 40 IIA, 41 IIB, 14 IIC	Biopsy	Compare GEP to AJCC in predicting 5 yr RFS, distant mets, and OS	NR	NR	GEP+AJCC for Recurrence: 90/71/NR/NR, 3.10/0.14; Distant Mets: 88/63/NR/NR., 2.38/0.19; Death: 82/62/NR/NR, 2.16/0.29		(78)
Gene Expression	RT-PCR	qRT-PCR for PAX3d to monitor recurrent in stage II-IV disease	198	111 melanoma, 87 healthy controls	Plasma draw	Identify recurrence in stage II-IV disease with plasma PAX3d mRNA	NR	0.823	Stage II–III relapse: 67/75/67/75, 2.68/0.44; Stage IV relapse: 75/93/43/98, 10.7/0.27	Quantitative	(79)
Serum nucleic acid	Circulating tumor DNA	PCR of BRAF and NRAS mutants	125	20 with progression, 9 with pseudo-progression	Plasma draw	Differentiate radiologic pseudo-progression and true progression	NR	NR	90/100/100/82, >90/0.1	Quantitative	(80)
**Monitoring of treatment effect**											
Serum nucleic acid	Circulating tumor DNA	BRAF V600mut ctDNA by qRT-PCR	36	16 before therapy, 20 after therapy	Plasma draw	Monitor patient response to BRAF/MEK inhibition therapy with BRAF V600mut ctDNA	NR	NR	70/100/NR/NR., >70/0.30	Quantitative	(81)

AUC, area under (receiver operating characteristic) curve; CTC, circulating tumor cells; ctDNA, circulating tumor DNA; ELISA, enzyme-linked immunosorbent assay. FFPE, formalin-fixed paraffin embedded; GEP, gene expression panel; LR+, positive likelihood radio; LR–: negative likelihood radio; Mets, metastasis; NPV, negative predictive value; NR, not reported; OS, overall survival; PPV, positive predictive value; RFS, recurrence-free survival; S, sensitivity; qRT-PCR, quantitative real time polymerase chain reaction; SLNB, sentinel lymph node biopsy; Sp, specificity.

‡ *All LR values are calculated based on reported sensitivity and specificity*.

#### Prognostic Biomarkers

Prognostic biomarkers compose the broadest category of biomarkers. In terms of clinical decision impact, they can range from nebulous indicators of survival odds to potential heralds of disease recurrence. The principal issue with survival indicators is that they may require adoption by the AJCC or other analogous staging protocols prior to widespread utilization. Otherwise, the ordering clinician may be unsure how an unfavorable result will affect their patient's stage, choice of intervention, and ultimately outcome. A biomarker with excellent test characteristics may therefore be effectively useless if it is not incorporated into a staging framework from which clinical decisions can be made.

One commercially available prognostic test is the Castle DecisionDx assay. While the DecisionDx assay is not included in AJCC staging nor NCCN treatment guidelines, the test aims to identify patients with stage I and II disease who may be at increased risk of metastasis and death. Analytically, this gene expression profile (GEP) has demonstrated reproducibility across instruments in separating “class 1” (low-risk) from “class 2” (high-risk) probability scores and in technical concordance between runs (82). Results from small retrospective and prospective clinical cohorts have suggested improved ability to stratify risk of relapse and distant metastasis independent of SLNB status (83–87). However, each of these studies are limited by the lack of stage or substage breakdown of test performance, rendering it difficult to determine how much additional information is imparted by the biomarker beyond existing staging criteria. Podlipnik et al. studied the additive utility of DecisionDx probability scores in conjunction with combined AJCC stages (“low-risk”: IB-IIA, “high-risk”: IIB-IIC) and found disease free survival was lower in melanoma patients with DecisionDx high-risk “class 2” scores, regardless of grouped AJCC staging (87). Furthermore, in a 205 patient cohort, comprised of combined Stage I and II melanoma cases, where the DecisionDx GEP was combined with the AJCC Individualized Melanoma Patient Outcome Prediction Tool, the authors reported improved sensitivity but worse specificity for predicting disease recurrence, distant metastasis, and death compared to either metric alone (78). However, because neither positive predictive value/negative predictive values (PPV/NPV), nor AJCC substage breakdown of DecisionDx test performance were reported, it is challenging to determine the utility of this test beyond AJCC staging criteria, and it remains to be determined if and how this test can be integrated into current standard of care.

The AJCC melanoma staging and substaging criteria are built upon robust collection of patient clinicopathologic features and have been refined over decades using long-term outcomes data for more than 45,000 patients with melanoma (1). As such, this is the standard to which prognostic biomarkers must be measured, and full transparency of clinical characteristics and AJCC substages of patients in future studies will be necessary to meaningfully compare prognostic outcomes to AJCC staging (88, 89). Because of these limitations, a recent consensus group statement from the melanoma prevention working group members recommended against routine use of prognostic GEPs, including the DecisionDx, outside of clinical trials, without thorough discussion of testing limitations with the patient, or outside of multidisciplinary conference recommendations (5).

#### Stratifying Risk of Lymph Node Metastasis

**Figure 5:**
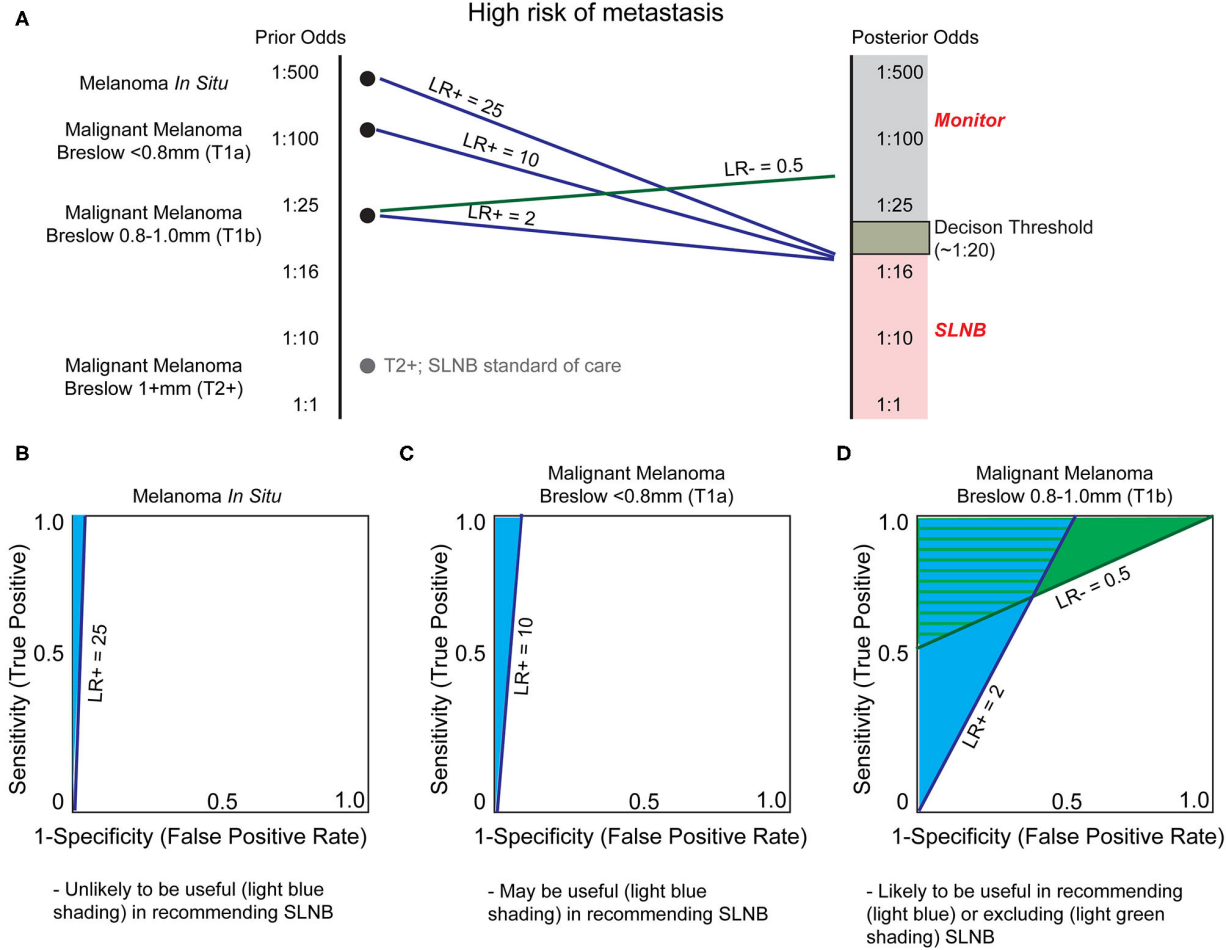
Utility of biomarkers to augment Breslow depth in determining which patients are offered sentinel lymph node biopsy (SLNB). **(A)** As depth of melanoma increases, from *in situ* to greater than one millimeter, the probability of sentinel lymph node positivity, and thereby stage and treatment selection, increases. Sentinel lymph node biopsy (SLNB) is positive in ~5% of patients with T1b melanoma, resulting in a decision threshold posterior odds of ~1:20 for many surgical oncologists to offer SLNB and reflecting NCCN guideline recommendations (90). As SLNB is not routinely offered for melanoma *in situ* or T1a melanomas, the incidence of SLNB positivity is unknown. We therefore estimate that these are substantially and progressively less positive as tumor thickness decreases. SLNB is standard of care for T2+ melanomas (gray dot) and thus no biomarker is likely to alter this recommendation without exceptionally low LR- test characteristics. **(B)** Currently patients with melanoma *in situ* are not generally offered SLNB since the likelihood of positivity (prior odds) is very low. A very high LR+, estimated at 25, would be necessary to recommend SLNB. **(C)** As the melanoma Breslow depth increases, the prior odds of sentinel lymph node metastasis increases (becomes closer to 1:1). Therefore, a lower LR+, such as LR+ ≥10, would be necessary to prompt SLNB. **(D)** Biomarkers may have the greatest utility for T1b malignant melanomas. Current NCCN guidelines recommend thoughtful consideration of SLNB based on patient-specific factors, and any test that could offer relatively small changes in certainty could be of great clinical utility. Thus, a modest LR+ of 2.0 or LR– of 0.5 may be sufficient for a clinically-actionable biomarker.

A major clinical decision point is to perform a sentinel lymph node biopsy (SLNB) for patients with T1b tumors or in AJCC stages IB and II, which will upstage the patient to stage III if positive (Table 1). At present, the benefit of SLNB is in determining who is eligible for adjuvant systemic therapy (90). Examples of adjuvant therapy include the combination of dabrafenib plus trametinib in resected Stage III BRAFV600E/K mutant melanoma, which improves 5 year overall survival (91), and pembrolizumab which improves relapse free survival in high risk, resected, Stage III melanoma patients (92). Since current NCCN guidelines recommend SLNB if the odds of a positive node are >5% (90), the positive rate of SLNB is low (93). In theory, there may be significant clinical utility for risk-stratifying biomarkers to determine which patients may benefit from this procedure (Figure 5). Because of the large degree of uncertainty as to which patients will benefit from SLNB, minimal changes in LR of LN metastasis may be sufficient to alter clinical decision making in T1b melanomas (Figures 5A,D). Further, the risk to the patient of an undetected LN metastasis (a missed opportunity to treat Stage III disease before progression) must be incorporated into the decision. Indeed, improved efficacy of adjuvant systemic therapy alters the risk-benefit analysis of SLNB, potentially shifting the decision to favor surgery in some patients.

In theory, a prognostic assay with high LR- or NPV could rule out SNLB eligible patients who would not benefit from the procedure. Conversely, a test with high LR+ or PPV could identify patients who may benefit from SLNB but otherwise would not have been offered (i.e., melanoma *in situ*; Figure 5B, or T1a melanoma; Figure 5C). In reality, NPV and PPV are often inversely related, and as such, the goals of the test must be clearly defined. For a molecular assay to replace the gold standard SLN procedure in patients without clinically positive nodes (94), which at present is the only determinant of patient eligibility for systemic therapy (90), the assay must have an NPV higher than the SLN procedure [NPV of SLNB reported up to 94% (95)]. While the value of the assay remains uncertain, SkylineDx has conducted validation studies for this indication. In their initial study, SkylineDx combined established prognostic factors, such as patient age and Breslow depth, with their 11 GEP to predict SLN metastasis (71), thereby convoluting the contribution of the GEP to the classification. Concerningly, the initial gene set was defined using a cohort of only seven patients—three with and four without regional metastasis (72)—calling into question the likelihood the GEP will perform well across a greater diversity of patients.

A recent consensus statement expressed concern about the lack of test performance metrics in SkylineDx's studies (5). These values have since been released (72), demonstrating a specificity of 53% and LR- of 0.24 for TIIa disease with otherwise unremarkable test characteristics (Table 4). Based on these results and our model (Figure 5), there is a narrow window of utility for determining which TIIa tumors would benefit from a SLNB and a larger range of utility in determining which patients could safely forego SLNB using this test. However, the combined low number of positive SLNB (128 of 754 biopsies) and small discovery cohort (*n* = 7) are concerning given the heterogeneity of melanoma. Extensive validation on diverse cohorts that report a NPV higher than SLNB are required to determine whether the SkylineDx GEP is capable of answering the clinical questions for which these tests are ordered.

Similarly, a 2019 Castle DecisionDx study claimed the 31 GEP assay distinguished individuals >55 years of age with T1-T2 disease who have a <5% chance of SLN involvement. However, no measures of accuracy or predictive value were presented, making it challenging to assess the clinical utility of the test for this application (70). Of greater concern, the Castle DecisionDx test was developed to assess for metastatic risk associated with a primary melanoma, not to predict SLN involvement. Although the repurposing of a clinical test developed as an indicator of one condition to another is possible, successful performance would be fortuitous, especially since previous validation studies assessing metastasis risk groups are irrelevant when considering this test's utility for assessing the value of SLNB. Thus, while the Castle DecisionDx test may eventually improve the NPV of SLNB in prognosticating relapse or distant metastasis (83–85), without further study this test should not be considered to supplant SLNB, which remains the most important prognostic staging element guiding further treatment (90).

To determine the impact of these GEPs, and other tests, on decision to biopsy SLNs and their role in staging (see below), a large retrospective study and/or longitudinal studies will be necessary to determine how these biomarkers predict clinically meaningful outcomes like distant metastasis free-survival and all-cause mortality. Meta-analysis of existing studies has demonstrated that currently available GEP tests have limited ability to prognosticate recurrence in early stage melanomas (96). Due to the cost of conducting such trials, in terms of economic considerations and irreplaceable patient samples, the melanoma community will need to identify and prioritize the coordinated validation of only the most promising biomarker assays. Considerations for test selection need to include not only test characteristics, but also the details by which those characteristics were generated (size and diversity of cohorts, independence of studies, selection bias) and the likelihood to change clinical decision making when considering current guidelines.

#### Monitoring for Disease Recurrence

Monitoring for disease recurrence via serial imaging is costly and, depending on modality, can lead to significant cumulative radiation exposure in patients. Use of blood- or serum-based biomarker assays (termed liquid biopsies) may be able to identify residual or recurrent disease prior to demonstrable evidence of such disease with imaging studies (97). Prediction or early identification of distant metastasis would enable clinicians to begin adjuvant systemic therapy prior to confirmation of macroscopic metastatic disease by radiology.

Current AJCC staging criteria and NCCN guidelines incorporate one serum-based prognostic biomarker in the treatment of melanoma: lactate dehydrogenase (LDH), a proxy for tumor lysis and disease burden (1, 90). Several studies have demonstrated that a baseline LDH level >2 times above the laboratory-established upper reference range is associated with poor survival outcomes in patients with stage IV melanoma, despite the advent of targeted immunological agents (98–101). However, due to lack of sensitivity and biological specificity for melanoma, LDH has been relegated to advanced metastatic disease staging and treatment effect monitoring (1, 90). Other studies have proposed biomarkers with test characteristics superior to LDH. These include U2 small nuclear RNA, which is reported to correlate with increased risk of regional (AUC 0.80) and distant (AUC 0.94) metastasis (74), and serum *PAX3d* mRNA which has potential to monitor recurrence in stage IV disease, with specificity up to 93% and NPV up to 98% (79). However, further prospective testing is necessary to determine if and how these tests could be incorporated into current treatment recommendations for the prediction of early metastasis.

In a proof-of-concept study, circulating tumor cells (CTC) quantification from peripheral blood was a specific indicator of peripheral metastasis with a PPV of 100% (73). Besides the small cohort size (*n* = 15), one major limitation of this study was the unusual combination of immunomagnetic cell sorting and four-color immunofluorescence microscopy. A second challenge for CTC approaches in general is the propensity for melanoma cells to de-differentiate and thereby down-regulate lineages-specific cell markers used for selecting CTCs (S100, MelanA, SOX10). If CTC quantification could be adapted to a more widely available and objective platform, such as flow cytometry, and invariant melanoma markers identified, then it might find utility as a routine liquid biopsy. Alternatively, plasma concentrations of proteins other than LDH, such as osteopontin and S100 (75), may have functionality in predicting early metastasis, but have yet to be validated.

Identifying and validating biomarkers for minimal residual disease burden (MRD) have the potential to reduce imaging frequency, assess post-treatment disease burden, and identify microscopic disease relapse that is undetectable with standard imaging. One promising assay type is the quantification of common melanoma mutations as circulating tumor DNA (ctDNA) from peripheral blood. While there are ctDNA assays approved to identify actionable mutations in a variety of solid tumor types, these assays usually have a limit of detection (LOD) between 0.1 and 1.0% of ctDNA, which corresponds to 1 tumor sequence per 100–1,000 normal sequences (102). Current molecular techniques including digital droplet PCR, allele-specific oligonucleotide primer PCR, and NGS have the ability to further increase sensitivity by detecting known mutant alleles, resulting in an LOD between 0.001 and 0.1% of sequences for hematologic malignancies such as multiple myeloma (103). Detecting solid tumor ctDNA in peripheral blood has been more challenging due to the lower estimated amount of ctDNA present in non-metastatic cancer patients (102), but recent advances using techniques such as targeted digital sequencing (TARDIS) have improved the LOD of tumor-specific sequences to 0.01% of circulating DNA (104), likely representing the absolute minimum sensitivity required to detect a 5 mm primary tumor in breast cancer (102). Since the amount of ctDNA in patients with melanoma is on average 2 orders of magnitude less compared to breast cancer (105, 106), it is expected that a more sensitive assay with a lower LOD will be required to assess MRD in melanoma.

Several pilot studies have assessed the use of ctDNA as a MRD marker to monitor disease progression and relapse, with a few achieving a competitive LOD. One example demonstrated that monitoring *BRAF* V600 mutant sequences could identify disease progression prior to radiographic evidence in 44% of cases and was co-identified with imaging in an additional 26% (sensitivity 70%, specificity 100%, LOD 0.01%) (81). Similarly, a second study examining the most common *NRAS* and *BRAF* mutants found that detectable levels of ctDNA did not occur in cases with radiologic pseudoprogression (sensitivity 90%, specificity 100%, PPV 100%, NPV 82%, LOD <10 copies/ml or ~1%) (80). The only study with a prospective validation cohort (*n* = 36) found that increasing mutant *TERT*/*NRAS*/*BRAF* ctDNA preceded radiologic evidence of progression by an average of 3.5 months (AUC of 0.85–0.87, LOD: BRAF^V600E^ and NRAS^*Q*61^ 0.01%, TERT^C250T^ 1 copy/reaction and TER^C228T^ 10 copies/reaction) and were superior to LDH and S100 serology for this indication (107). Additional studies are needed to confirm these findings in a larger cohort, to determine the impact of observed mutational heterogeneity on test performance (107), and to find surrogates for melanomas that do not bear a *TERT, NRAS*, or *BRAF* mutation.

### The Wild West of Predictive Biomarkers for Systemic Therapy

For Stage III melanomas that have metastasized to sentinel lymph nodes and for those in which nodal or in transit metastatic disease has been surgically resected, adjuvant therapy using anti PD-1 immunotherapy or BRAF/MEK inhibition is currently recommended for consideration based on recent clinical trial data showing improved relapse-free survival (RFS) (91, 108). There is significant interest in using adjuvant therapy in Stage II high-risk melanomas, as evidenced by a large, double-blind, prospective, randomized clinical trial currently evaluating the safety and efficacy of immune checkpoint blockade in resected Stage II melanoma (KEYNOTE-716: https://clinicaltrials.gov/ct2/show/NCT03553836). Recently an 11-gene GEP was published that demonstrated the ability to stratify patients with stage II melanoma into high GEP score groups and low GEP score groups, with the latter having significantly higher melanoma specific survival and relapse free survival rates (109). The authors of this study speculate that patients with higher GEP scores may be better candidates for adjuvant therapy due to the increased risk for relapse and death, potentially reflecting ambitions to test this hypothesis in a future study (109).

If an actionable *BRAF* V600 mutation is identified by sensitive and specific molecular testing (110), then BRAF plus MEK inhibitor therapy is an option for first-line therapy in resected Stage III and in Stage IV melanoma patients (90). Regardless of BRAF mutation status, immune checkpoint inhibitors (ICI) against PD-1 and CTLA-4 are frequently used due to improved progression-free and overall survival compared to both chemotherapy and combined BRAF/MEK inhibition (111–113). However, ~65% of Stage IV metastatic melanomas do not significantly respond to ICI therapy (114–116), indicating an unfilled niche for future biomarkers to discriminate responders from non-responders prior to therapy initiation. Furthermore, accurately predicting which Stage III, Stage II, and even Stage I patients, who comprise the majority of cases of melanoma specific mortality (117), have the potential to benefit from adjuvant therapy is a major goal of predictive biomarkers in melanoma.

With the development of new ICIs, kinase inhibitors, combination therapy regimens, and other novel treatment modalities, there will be an increasing need for biomarkers to match patients to optimal adjuvant therapies. Successful predictive biomarkers will have the potential to decrease patient morbidity by avoiding side-effects of less helpful treatments, boost response rates by identifying the most appropriate therapy upfront, and increase the cost-effectiveness of treatment by avoiding expensive ICIs when not indicated. When there are multiple treatment options considered first- or second-line treatments, there is even more value in the role of biomarkers guiding therapy selection, however the dichotomous framework that we have presented for other key decision-making points in the treatment of melanoma is less applicable. The complex decisions surrounding adjuvant therapy selection would necessitate multi-dimensional LR+/LR- analyses not attempted in this review due to exponentially increasing complexity. We await the results of ongoing studies and clinical trials in this field and refer the reader to several recent reviews that cover the impressive breadth of proposed biomarkers for use with ICI (118–121).

## Discussion

Many studies have addressed the potential utility of biomarkers in the diagnosis and treatment of melanoma, highlighting the drive to improve melanoma patient care through novel technologies. In this review, we have discussed several key clinical decision points in the diagnosis and treatment of melanoma including determining when to biopsy, aiding in the histopathologic diagnosis of melanoma, and stratification of prognosis of malignant melanoma. We have applied fundamental theory of clinical test utility to model these decision points and identify test characteristics required for a biomarker to influence decision making. This discussion is not intended to be an ultimate determination of the utility of any specific biomarker but rather to highlight how clinical-use-assumptions affect the characteristics required of a test to be potentially incorporated into practice. We hope this framework and analysis will assist basic science researchers in designing investigations with a greater likelihood of true translation and aid clinical researchers in identifying which candidate biomarker tests are worth allocating resources for validation.

Similar to other malignancies, melanoma is best treated when diagnosed early. When a melanoma is identified prior to invasion, treatment has high rates of cure with wide local excision alone (1, 122) but as invasion depth increases toward eventual metastasis, treatment becomes increasingly morbid and survival rate decreases. An important observation that arises from the modeling applied here is that across this spectrum, the characteristics of an ideal biomarker for melanoma change to reflect the changing clinical considerations surrounding treatment morbidity and efficacy. For early melanomas (T0-T1a), specificity (and thus LR–) is emphasized for diagnosis to avoid missing melanomas that are treatable with excision alone. At this stage of diagnosis, an intermediate lesion will default to treatment given the relatively low morbidity of excision, and as a result, sensitivity is generally less important. However, for primary malignant melanomas with a more significant invasive component (T1b-T4), the question of staging and assessing SLNB utility becomes important. Here the utility of a biomarker with high sensitivity (LR+), specificity (LR–) or both become quite useful. With Stage III+ melanomas demonstrating metastatic behavior, biomarkers with optimized specificity characteristics (LR+) suggesting response to specific treatment regimens will potentially be more useful given the higher morbidity and cost associated with adjuvant therapies.

Ideal biomarkers would prioritize neoplasia with true malignant potential for appropriate treatment, while providing confidence that intermediate neoplasia with reassuring biomarker findings can safely avoid further treatment. However, due to a lack of an ideal gold standard for verification, rigorous clinical validation of candidate tests by conventional standards is difficult for ambiguous lesions. Practical considerations of cost, ease of use, and tissue requirement will also factor into which biomarkers can be incorporated into clinical practice. Thus, the field should take great care when considering which biomarkers to prioritize for validation. While immunohistochemistry, copy number variation, and FISH represented the mainstay of biomarker use in this space for many years, more recently reported techniques including gene expression profiling, DNA methylation, and ciliation index have the potential to provide superior test characteristics and objective interpretation at lower cost and tissue requirement and thus represent exciting new potential avenues for further improvement in the diagnosis of melanoma (44, 52, 60).

The challenges of developing novel biomarkers for use in melanoma diagnosis are significant. One of the most substantial obstacles is the inherent subjectivity of histopathologic diagnosis of melanoma. When validation studies rely on histopathologic diagnosis, ambiguous lesions are often excluded, though these are the exact cases most in need of adjudication with an objective biomarker. Because of the imperfect nature of this gold standard, several studies have used consensus diagnosis from three or more experienced dermatopathologists (45, 46), which we would recommend of future studies. At a minimum, reporting the number of pathologists diagnosing each case, and whether or not they are experts in pigmented lesions, is helpful to determine the reliability of the study. Given the subjectivity of histopathologic diagnosis, we should strive to review the performance of biomarkers compared to long-term patient follow up data, despite the large sample sizes, cost, and time necessary to conduct such studies. Recurrence-free survival and melanoma-specific survival are perhaps the true gold standards, and gathering these data is essential to incorporate biomarkers into the standard of care and meaningfully improve patient outcomes.

Because of the high level of uncertainty surrounding the determination of which patients will benefit from SLNB, relatively small changes in certainty imparted by a biomarker may result in large changes in clinical management. The models applied here suggest that biomarkers with high LR+/sensitivity characteristics have the potential to confirm the utility of SLNB, while biomarkers with high LR-/specificity characteristics may prove useful in determining which patients can safely forego SLNB. Due to the high cost and morbidity associated with SLNB compared to wide local excision alone, and the potential for biomarkers to prove clinically useful with either high LR+, LR– or both, significant attention has rightfully been paid to biomarkers that address the question of which patients should undergo SLNB. Compared to diagnostic biomarkers, those targeting the necessity of SLNB require a relatively smaller change in certainty (smaller LR+ or LR–) to be clinically actionable. This, coupled with the costs of SLNB, financial and otherwise, suggest that biomarker tests in this space are worthy of the significant resources needed for further development.

Regardless of the purpose of a candidate biomarker, large prospective validation studies conducted on diverse and relevant patient populations that demonstrate clinically meaningful outcomes over time are needed to propel biomarkers into the mainstay of treatment. The time, large sample sizes, potential coordination across multiple trial sites, and significant expense of such studies present major barriers to generalizable validation studies. Because of these challenges, the vast majority of published biomarkers do not have multiple validation studies. Even those that do (such as Myriad myPath Melanoma and Castle Biosciences DecisionDx-Melanoma) (44, 76) have yet to be incorporated into NCCN guidelines due to lack of evidence in ambiguous melanocytic neoplasms and lack of evidence of actionable prognostic information across the melanoma spectrum (90). Due to these lengthy development and validation phases, combined with the pressing need for clinical tests, novel biomarkers are sometimes ordered by clinicians prior to robust establishment of clinical utility (35, 123). It is generally recommended that clinical tests should not be ordered that do not have the potential to change management or prognosis. Unnecessary testing can result in unanticipated results that complicate an otherwise straightforward diagnosis, cause undue physical and emotional hardship on the patient, lead to additional unwarranted testing, and increases costs borne by the patient and medical infrastructure (124–127).

## Conclusions

The challenges of clinically validating a biomarker are significant. Given the substantial resources necessary to comprehensively validate a biomarker, clarity regarding the clinical question being addressed and the practical considerations that would confer clinical utility should be fundamental considerations prior to initiating a study. This review applies the fundamental theory of biomarker utility to estimate the necessary test characteristics required to influence clinical decision making in the diagnosis and treatment of melanoma, and summarizes the test characteristics and rigor of existing and candidate tests. It is clear that there is significant room for improvement in the validation of biomarkers in this space, and we hope that the models and considerations summarized here will help guide the community in study design and investment that will yield the maximum clinical utility.

## Author Contributions

DD and RJ-T conceptualized this work. DD, ES, and RJ-T performed review of the literature and wrote the manuscript. All authors contributed to the article and approved the submitted version.

## Conflict of Interest

RJ-T is an inventor on international patent application no. PCT/US2019/023834 concerning the use of microRNA ratios as molecular diagnostics for melanoma. The remaining authors declare that the research was conducted in the absence of any commercial or financial relationships that could be construed as a potential conflict of interest.
